# Pedunculated osteochondroma of right humerus

**DOI:** 10.11604/pamj.2026.53.64.48298

**Published:** 2026-02-06

**Authors:** Amit Toshniwal, Dhairya Veragiwala

**Affiliations:** 1 Department of Respiratory Medicine, Datta Meghe Institute of Higher Education and Research, Wardha, Maharashtra, India,; 2 Department of Orthopedics, Datta Meghe Institute of Higher Education and Research, Wardha, Maharashtra, India

**Keywords:** Osteochondroma, bone lesions, chondrosarcoma

## Image in medicine

A 16-year-old boy presented with a painless, hard swelling over the lateral aspect of his right upper arm. There was no history of trauma, constitutional symptoms, or functional limitations. On clinical examination, the mass was firm and nontender. Radiography of the right upper limb revealed a solid, irregular outgrowth of the right humerus (panel A). Magnetic resolution imaging (MRI) revealed a bony outgrowth arising from the lateral aspect of the diaphysis of the right humerus, suggestive of a pedunculated osteochondroma (panel B). Owing to cosmetic concerns and the potential risk of neurovascular compression, surgical excision was performed. Histopathological examination confirmed a benign osteochondroma with no evidence of malignant transformation. The postoperative radiography revealed cosmetic improvement (panel C) and the patient remained asymptomatic. This case underscores the importance of recognising osteochondroma as a common benign entity in adolescents, while also considering the need for surgical intervention in symptomatic or cosmetic cases. Early imaging and appropriate management can prevent complications, particularly in lesions with atypical growth or proximity to vital structures.

**Figure 1:**
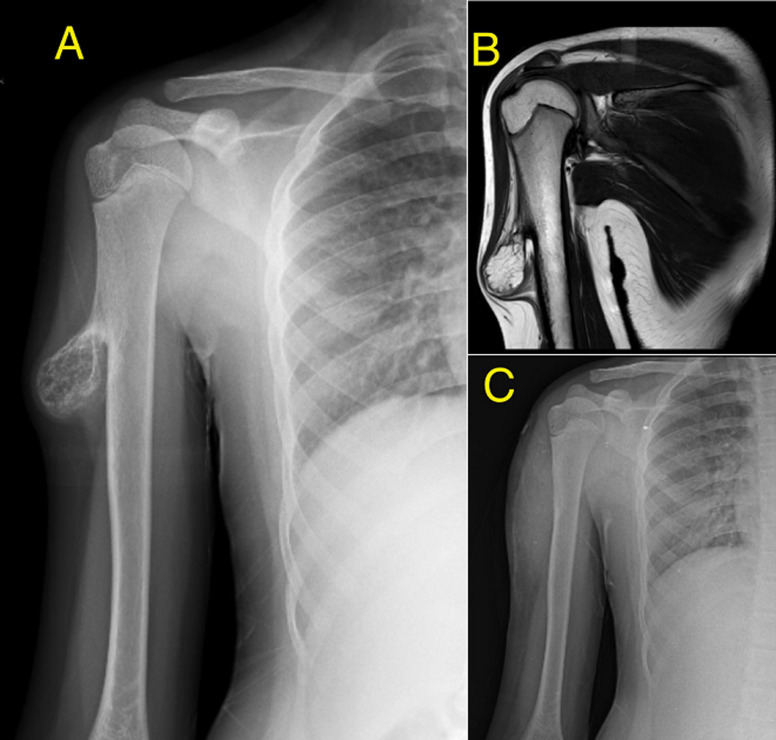
A) right humerus showing irregular outgrowth; B) T2-weighted MRI of right humerus showing bony outgrowth arising from the lateral aspect of diaphysis; C) postoperative radiography showing cosmetic improvement

